# Developing nurse medication safety training in a health partnership in Mozambique using behavioural science

**DOI:** 10.1186/s12992-017-0265-1

**Published:** 2017-07-04

**Authors:** Eleanor Rose Bull, Corina Mason, Fonseca Domingos Junior, Luana Vendramel Santos, Abigail Scott, Debo Ademokun, Zeferina Simião, Wingi Manzungu Oliver, Fernando Francisco Joaquim, Sarah M. Cavanagh

**Affiliations:** 1 University of Manchester, Manchester, UK; 2 NHS Grampian, Aberdeen, UK; 3 Beira Central Hospital, Beira, Mozambique; 4 Ipswich Hospital NHS Trust, Ipswich, UK; 5 East Anglia Medicines Information Service, Ipswich Hospital NHS Trust, Ipswich, UK; 6 University of Suffolk, Ipswich, UK

**Keywords:** Patient safety, Drug dosage calculations, Pharmacist, Administration and dosage, Medication errors, Global health, Education, nursing, continuing, Behavioral sciences, Behavioral medicine

## Abstract

**Background:**

Globally, safe and effective medication administration relies on nurses being able to apply strong drug calculation skills in their real-life practice, in the face of stressors and distractions. These may be especially prevalent for nurses in low-income countries such as Mozambique and Continuing Professional Development post-registration may be important. This study aimed to 1) explore the initial impact of an international health partnership’s work to develop a drug calculation workshop for nurses in Beira, Mozambique and 2) reflect upon the role of health psychologists in helping educators apply behavioural science to the training content and evaluation.

**Methods:**

In phase one, partners developed a training package, which was delivered to 87 Portuguese-speaking nurses. The partnership’s health psychologists coded the training’s behaviour change content and recommended enhancements to content and delivery. In phase two, the refined training, including an educational game, was delivered to 36 nurses in Mozambique and recoded by the health psychologists. Measures of participant confidence and intentions to make changes to healthcare practice were collected, as well as qualitative data through post-training questions and 12 short follow-up participant interviews.

**Results:**

In phase one six BCTs were used during the didactic presentation. Most techniques targeted participants’ capability to calculate drug doses accurately; recommendations aimed to increase participants’ motivation and perceived opportunity, two other drivers of practice change. Phase two training included an extra seven BCTs, such as action planning and further skills practice. Participants reported high confidence before and after the training (*p =* 0.25); intentions to use calculators to check drug calculations significantly increased (*p =* 0.031). Qualitative data suggested the training was acceptable, enjoyable and led to practice changes, through improved capability, opportunity and motivation. Opportunity barriers to medication safety were highlighted.

**Conclusions:**

Reporting and measuring medication errors and related outcomes is a complex challenge affecting global efforts to improve medication safety. Through strong partnership working, a multi-disciplinary team of health professionals including health psychologists developed, refined and begin to evaluate a locally-led drug calculation CPD workshop for nurses in a low-resource setting. Applying behavioural science helped to collect feasible evaluation data and hopefully improved impact and sustainability.

## Background

A crucial element of the role of hospital nurses across the world is to correctly calculate doses and safely administer medication to patients. In most cases, administering medication has the desired, beneficial effect and improves the patient’s health and wellbeing. However, sometimes problems arise, due to errors in prescribing, dispensing, or calculating doses, or through incorrect or omitted administration, which can result in serious patient harm. [1] In the UK, improved reporting of medication incidents in recent decades enables NHS England to estimate that 1.8 million serious prescribing errors occur each year. [2–5] One observational study suggested preparation error rates of 26% and administration error rates of 34%. [6] The figure is likely to be similar if not higher in Mozambique and other countries in Sub-Saharan Africa, given medication and health professional shortages, and errors may have a greater impact on morbidity and mortality than in higher income countries. [7, 8] The World Health Organisation (WHO) recently launched a Global Patient Safety Challenge on Medication Safety, calling for all member countries to reduce avoidable medication-associated harm by 50% in the next 5 years. Yet, they also acknowledge the lack of routine data collection in low-income countries. [8] In such areas, measurement of medication errors, and of the effectiveness of initiatives to improve them, is a complex challenge in itself [9].

Improving medication safety requires changes at many levels of a hospital system, with clinical governance for supply, stock management, prescribing, preparation, dispensing, administering and monitoring. Drug dose calculations and administration are particularly difficult nursing tasks in a busy ward environment, and factors contributing to medication errors include interruptions and distractions, staff fatigue and stress, equipment problems, patient factors and poor communication from colleagues. [10] In addition, the WHO challenge specifically highlights poor training as a key cause of medication error. [8] The UK’s Royal College of Nursing recommend nurses attend periodic medicines handling and management refresher training post-qualification as one of their Continuous Professional Development (CPD) activities. [11] However, few countries in Sub-Saharan Africa have similar recommendations, and where such CPD does take place, it is unknown how effective this is.

In some countries, health psychologists are employed in healthcare settings, where they work with patients and healthcare staff applying a scientific understanding of behaviour and its psychological determinants (collectively known as ‘behavioural science’). Health psychologists view professional practice as a set of behaviours, and work with educators to design and evaluate health professional CPD which is likely to maximise practice change (rather than only raising ‘awareness’ or ‘knowledge’ levels). They also help educators evaluate training, either quantitatively or qualitatively. Where quantitatively measuring behaviour and outcomes is not possible, they may suggest measuring proximal psychological determinants of behaviour such as confidence.

In terms of effective CPD components, according to an increasingly well-evidenced framework of behaviour change for designing interventions called ‘COM-B’ [12, 13], Behaviour change (B) depends on three groups of psychological determinants. These are perceived Capability (C), Opportunity (O) and Motivation (M). Capability generally includes knowledge and skills, whereas opportunity is the ability to use these in practice (by overcoming challenges in the physical environment, norms and social pressure). Finally, motivation includes the training participant’s views of the costs and benefits of making a change to their practice and also the influence of previous habits and routines (*see also Byrne-Davis* et al. *in this issue*). Most training aims to improve capability to change participants’ practice, but health psychologists can assist educators to understand the content of their training and add in further behaviour change techniques (BCTs) [14] targeting the other important drivers of practice. [12] Health psychologists may also examine CPD delivery methods: systematic review evidence suggests that a mix of interactive and didactic delivery is most effective [15] and that educational games can be an engaging and useful delivery method [16, 17]. Interaction and educational games may encourage active learning and practice, encouraging deeper mental processing meaning participants may be more likely to remember and use new information in practice. [18] Despite the potential gains in using behavioural science, this has not been applied to developing medication safety training in a resource-poor setting, nor in the context of a UK-African health partnership where health psychologists can work with other health professionals and educators to build sustainable CPD models.

This article describes the efforts of such a multi-disciplinary health partnership to develop and then refine a medication safety CPD workshop in Beira Central Hospital, Mozambique, using behavioural science. Despite rapid growth in the last 20 years, and great expansion to its healthcare workforce, access to healthcare in Mozambique remains a challenge and it has one of the lowest country rankings in the human development index (181 of 188 countries), [19] making it a priority area for partnership work to improve health [20]. The Ipswich-Beira NHS Health partnership is a UK government funded Health Partnership, supported by the Ipswich Hospital NHS Trust and currently administered by the Tropical Health and Education Trust. The two hospitals have a long-standing link working on many projects to strengthen health systems and improve patient safety in Beira Central Hospital. This has involved pharmacists, doctors, nurses and equipment maintenance engineers from the UK and Mozambique and, more recently, health psychologists from The Change Exchange programme (*see Byrne-Davis* et al. *in this issue*). One strand of the partnership’s work since 2014 has been medication safety, [21] including interventions such as developing medication error reporting, stock management systems, ward security and most recently, developing a drug calculation skills training CPD workshop for nurses.

This article aimed to 1) explore the initial impact of the drug calculation CPD workshop, in the context of the complexities of this important issue outlined above and 2) reflect upon the process of including behavioural science in a multi-professional health partnership. We hope to share learning with other partnership teams working to develop sustainable medication safety interventions in lower and higher income settings.

## Methods

### Aim and design

This research aimed to develop and refine a sustainable CPD training workshop for ward nurses on drug calculations. We applied recommended health psychology methods [12] using the COM-B model and a structured list of behaviour change techniques known as the BCT Taxonomy v1 [14] to refine and explore the initial impact of the intervention.

### Setting and participants

The health partnership is between Ipswich Hospital, East England, and Beira Central Hospital (BCH), a large referral and teaching hospital in Central Mozambique. The hospital serves over 8 million people with more than 1000 beds and 27,000 annual admissions. [22] Local figures suggest a current staff membership of 1800, including over 300 nurses of varying levels and training backgrounds. [Internal hospital data, personal communication July 2016]. Over the three times the drug calculation CPD workshop was delivered, a total of 123 nurse participants attended.

### Training development and delivery

The nurse medication safety CPD session was developed and refined during 2015 and 2016. For the purposes of this paper, we split these into two ‘phases’ of development.

#### Phase one

An initial needs assessment (including discussion with hospital nurses and pharmacists, and research on the HIFA Portuguese discussion forum) and an omitted doses audit was conducted by the partnership’s pharmacists which highlighted the potential need for drug calculation training. Ipswich partners shared a drug calculations CPD training package used both at Ipswich Hospital and the University of Suffolk with student and qualified professionals. This included a PowerPoint presentation with information on how to calculate doses, infusion rates, dilution of injectable medicines including the correct choice of diluent, unit dose conversions and other commonly encountered difficult drug calculations. The Beira partners adapted this for local relevance (e.g. locally-available drugs) and translated it into Portuguese, Mozambique’s national language. The lead Beira pharmacist then initially delivered this twice, to 57 and 30 BCH hospital nurses respectively. The two health psychologists in the partnership observed the latter session during a visit of the UK partners to Beira and separately coded its behaviour change content using the BCT taxonomy v1 [14] and COM-B model. [12] Inter-rater agreement between the two coders was 99% indicating high coding reliability: the one disagreement was resolved during a follow-up meeting.

#### Phase two

The health psychologists met with partnership members from the UK and Mozambique to share results of their observations (see Results: Phase One) and discuss recommendations to incorporate further behaviour change techniques, to refine the content to maximise its impact. These were also disseminated in a report. The UK partners then investigated impactful ways to deliver the recommended additional BCTs. This included contacting Focus Games, [23] a UK company specialising in educational board games as a teaching tool for health professionals, who kindly donated two copies of their popular Drug Round Game to the partnership. In the game, teams of healthcare staff progress along a snakes and ladders board by taking turns to answer questions from the other team regarding drug calculation and general questions about medication safety. Partnership members translated game questions and answers into Portuguese. At the next opportunity, the partnership pharmacists piloted the Drug Round Game with senior pharmacy and nursing colleagues, before deciding to incorporate it in the CPD workshop in the next delivery, as part of a wider training day being organised by the partnership for nurses. The lead Beira pharmacist also made changes to the PowerPoint presentation in line with the health psychologists’ recommendations. The resulting two hours refined training package was delivered, again facilitated by the lead Beira pharmacist, to 36 further Beira staff nurses over 2 days. It consisted of the refined PowerPoint slide section with group discussion for one hour and small group interaction facilitated by pharmacists for the remaining hour through playing The Drug Round educational board game. Instructions were provided verbally by two translators, and interactions between participants in each game were translated for English-speaking partnership members. The training package was delivered during two consecutive days, and following feedback from the hospital nursing director who observed the training, the game play was adjusted to only include calculation questions (excluding the more general questions which were less relevant to the Mozambique context). The pharmacists were encouraged to become ‘floating facilitators’ to enable participants more independence and coaching when needed. Participants were also asked to practise using the calculators provided by the partnership. Again the health psychologists observed the BCTs and related components in the COM-B model that were delivered by the partnership’s educators.

### Data collection and analysis

No formal competence assessment was obtained to help evaluate the session in phase one. Beira partners felt that a pre-post competency ‘quiz’ used in the UK training would be viewed negatively by participants who may be concerned about possible disapproval or even disciplinary consequences if they made an error. However, evaluation was introduced in phase two.

It would have been ideal to evaluate the impact of the revised training through measuring behaviour (e.g. reported medication errors or omitted doses on drug charts), or outcomes (e.g. preventable morbidity and mortality). However, as discussed, robust systems and cultures of reporting do not yet exist in many low-income countries and these were ‘works in progress’ in other strands of the partnership’s work.

Instead, in terms of quantitative methods, during the game the facilitators unobtrusively counted the number of correct and incorrect responses to questions from teams to provide some informal assessment of competence. The health psychologists also suggested assessing participants’ confidence and intentions, since these are proximal psychological determinants of behaviour [24, 25]. Participants were asked two questions pre and post training (in Portuguese): 1) ‘do you feel confident to correctly calculate drug doses?’ and 2) ‘will you use a calculator the next time you calculate a drug dose, to make sure it's correct?’ to ascertain intentions. Following a pilot phase of the questions, a ‘yes’ or ‘no’ binary format was agreed upon. Participants were asked to respond anonymously to the two questions on paper and deposit responses into a box. Pre-post comparisons of the number of participants self-reporting as confident and with positive intentions were compared using SPSS (version 20) using two McNemar’s tests, where a significance value of *p* < .05 was applied.

Additionally, qualitative evaluation methods were employed in phase two, both at the end of the CPD workshop and in the following week. At the end of the training session, participatnts were asked ‘what did you think of the training?’ ‘what did you enjoy most?’ or ‘write down one thing you will do differently in your job because of this training,’ and asked to write this down anonymously and place into a sealed box. Two partnership members then conducted short follow-up interviews with an opportunity sample of 12 nurse participants the following week after attending the revised training workshop. The follow-up semi-structured interview questions related to the full training day not only the drug calculation CPD workshop, therefore a sub-sample of the analysis is presented here. Participants were asked open questions surrounding their experiences of the CPD workshop and how they were getting on putting their learning into practice, including barriers and facilitators they were experiencing. Field notes of responses were analysed by the health psychologists using the five steps of Framework Analysis [26] applying the tenets of the COM-B model. Framework Analysis is a useful method for research with specific questions, a short time frame and where a theoretical structure can be usefully applied [27].

### Ethical considerations

The study was granted local hospital board approval in lieu of a local research ethics committee and correspondence with the national ethics board confirmed that it did not meet criteria for national review, since the data presented were collected from staff as part of an evaluation of the CPD workshop and no data were personally identifiable or sensitive. Agreement to participate in the training was indicated by participants attending and signing into the training register; participants were assured that providing anonymous feedback and ratings for course evaluation was optional. Numbers of correct responses in the game were totalled at a whole group level; participants were not specifically informed of the tally to avoid causing stress, important given the perceived pressure to avoid mistakes discussed previously. However, it is inherent to the game is that teams with more correct answers have more chances to roll the dice and progress and facilitators made encouraging comments at times such as ‘well done, that’s three right in a row for your team!’. It was therefore obvious that facilitators and participants alike were aware of numbers of correct scores. The anonymous data were stored securely on a password protected iPad.

## Results

Phase One**:** The health psychologists observed six main BCTs in use in the the first version of the training workshop, mainly delivered didactically. These were techniques which generally work by building participants’ capability and to a lesser extent, motivation. Table 1 highlights these.

**Table 1:** BCTs observed in phase one training, delivery mode and link to components in the COM-B model

COM-B factor	BCTs to address this^1^	Example and how delivered in phase 1 (didactic or interactive)
Capability	4.1 Instruction on how to perform behaviour	Didactic**:** Facilitator and study author FJD talked through the steps needed to calculate an infusion rate.
	6.1 Demonstration of behaviour	Didactic**:** Examples given as step-by-step formula calculations.
	8.1 Behavioural practice and rehearsal	Interactive**:** Group asked to individually calculate in their heads answers to questions posed.
Opportunity		
Motivation	5.1 Information about health consequences	Didactic**:** FJD highlighted some health consequences of medication errors.
	9.1 Credible source	FJD was the lead pharmacist in the hospital and therefore a persuasive and perhaps motivating source about medication safety.
	13.2 Framing/reframing	Somewhat interactive**:** Through a question to the group, FJD emphasised that all hospital staff need to know this important information, emphasising the importance of multi-disciplinary approaches to medication management.

^1^BCT labels taken from Michie et al. [14]

The lead pharmacist reported that recruiting and engaging staff in the phase one training had been a challenge, with some staff talking of their high perceived capability in their drug calculations and reporting that they did not need training. This motivation barrier to attending training is also common in UK health professionals [28] and research suggests healthcare professionals tend to overestimate their perceived capability when performance is measured. [29, 30] Informal discussions with participants suggested that they felt the session was a valuable refresher and several participants commented that it would be easy to implement in practice. However some participants highlighted opportunity barriers to implementation, for example calculators were not available on the wards.

The health psychologists made the following evidence-based recommendations for phase two of the programme:

- Inclusion of some interactive delivery elements of training to enable active learning to take place and further practice, rehearsal and feedback that could help strengthen capability**.**
- Addition of BCTs targeted at use of opportunities: shaping the physical and social environment to be more conducive to accurate drug calculations (e.g. provision of calculators, engaging in action planning of how participants will use skills in their real-life busy practice).
- Addition of BCTs to build on motivation and help participants understand the importance of CPD and accurate drug calculation behaviours, such as feedback of local audit data highlighting drug error issues and use of interactive activities to help engage participants.

Phase Two: Following the revisions to the workshop, the health psychologists observed seven extra BCTs (thirteen in total), including a greater number addressing opportunity and motivation, and changes in delivery towards additional interactive elements, in Table 2.

**Table 2:** BCTs observed in phase two training, delivery mode and link to components in the COM-B model

COM-B factor	BCTs to address this^1^	Example and how delivered in phase 2 (didactic or interactive)
*Capability*	4.1 Instruction on how to perform behaviour	*Interactive and didactic:* Pharmacist facilitator explained in initial slides how to use international units, then participants instructed others during the game.
	6.1 Demonstration of behaviour	*Interactive:* Teams demonstrated their drug calculations to each other.
	8.1 Behavioural practice and rehearsal	*Interactive:* During the game, repeated practice in calculating drug doses.
*Opportunity*	*12.5 Adding objects to the environment*	The partnership provided calculators, pens and notepads to participants and encouraged their use to calculate accurately and show their workings.
	*1.1 Goal setting* and *1.4 Action planning*	*Interactive:* Time was allocated for participants to set goals and make a specific action plan about where and when they would use their calculator.
	*1.2 Problem Solving*	*Interactive:* Whole group discussion on difficulties distinguishing long and short-acting insulin and strategies to help, supported by lead nurse.
*Motivation*	5.1 Information about health consequences	*Didactic:* FJD highlighted some health consequences of medication errors.
	9.1 Credible source	As in phase one, the facilitator was a senior pharmacist in the hospital and therefore a persuasive and perhaps motivating source about medication safety.
	13.2 Framing/reframing	*Interactive:* Large group discussion, lively debate and team working in the game may encourage a new perspective, that it is acceptable to ask nursing and pharmacy colleagues for help in drug calculations.
	*1.6 Discrepancy between current behaviour and goal*	*Didactic:* Though a series of whole group questions, pharmacist jokingly pointed out a discrepancy between participants’ perceived and actual competence, highlighting the need to engage in the training.
	*1.9 Commitment*	*Interactive:* The health psychologists asked participants to share their action plan with a neighbour, to promote commitment to accurate drug calculation.
	*2.2 Feedback on behaviour*	*Didactic + Interactive:* The pharmacist facilitator included local audit data to PowerPoint slides as hospital-level feedback on drug calculation errors and outcomes. Also in the game, participants fed back to each other whether their drug calculations were correct.

^1^BCT labels taken from Michie et al. [14] Italic BCT labels = technique only observed in phase two

Partnership members observing the game noted that once participants understood the game’s rules they tended to be highly engaged and enthused, laughing and contributing to group solutions, asking for advice from pharmacist facilitators and using the calculators provided to accurately calculate doses.

### Quantitative evaluation measures in phase two

**Fig. 1:**
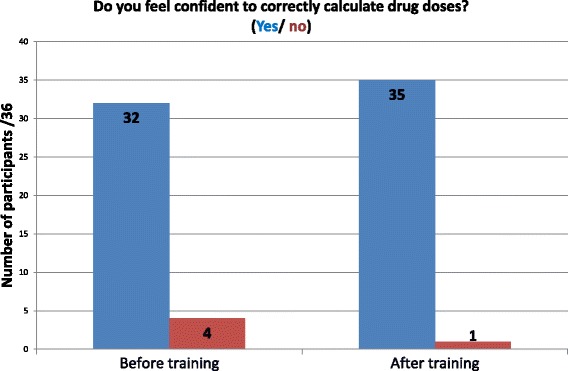
Do you feel confident to calculate drug doses? (yes/no). Participant responses to confidence question regarding drug calculations

**Fig. 2:**
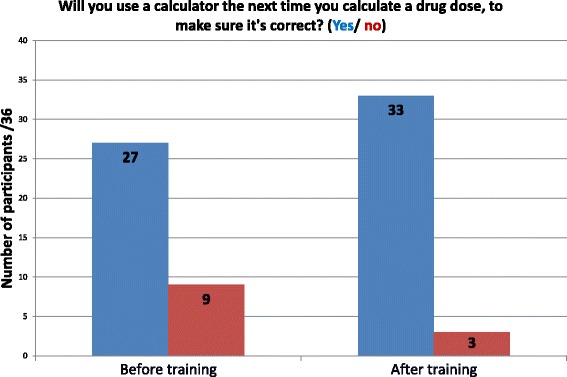
Will you use a calculator the next time you calculate a drug dose, to make sure it’s correct? (Yes/ no). Participant responses to intention question regarding using calculators for drug calculations

In total teams answered 16/22 questions correctly during the game in the revised CPD workshop. Numerical responses to the confidence and intention questions are presented in Figs. 1 and 2. Participants reported high confidence in their drug calculation skills both before and after the refresher training; intentions to use a calculator increased after the session. An exact McNemar’s test indicated a statistically significant difference in the proportion of nurses reporting intentions to use a calculator after the training session (*p* = 0.031), however there were was no statistically significant difference in relation to confidence (*p* = 0.25).

### Qualitative evaluation measures in phase two

In response to feedback questions immediately following training, the 49 comments received all indicated the training had been positively received: “*the training game about drug dose was really good, because it opened the mind more and so we will remember it always”* and “*I liked the drug calculations game, it was fantastic”.* Implementation planning of the training was also commented on, “*after this update I will pay more attention to how to calculate drugs. I will always use the calculator to calculate drug maths and ensure the right dose is prescribed”.*

In relation to the semi-structured interviews conducted in the week following the training, a summary of the COM-B model components and related themes are outlined in Table 3.

**Table 3:** Training participants’ views of the impact of the drug calculation CPD workshop on capability, opportunity, motivation and medication safety behaviours

COM-B Model Component	General Themes
Capability	• Increased knowledge to calculate drug doses • Calculators used for complex calculations
Opportunity	• Staffing problems leading to time shortages can impact on perceived opportunity for application of training
Motivation	• Increased awareness of the importance of accurate drug calculations and potential consequences of inaccurate drug calculations
Behaviours put into practice	• Changes to complex calculations – using calculator and/or adaptations to calculation approach

Nearly all participants reported that the drug calculation training had already impacted positively on their medication safety practices (behaviours), including calculating drug doses with more precision, using a calculator and informing colleagues on wards of information gained from the training. One participant reported *“I have used my calculator to confirm calculations, for example converting crystalised penicillin to millilitres”.* Another had advised his colleagues to “*always have a calculator by your side, don’t just rely on your mind to get the right answer”* and another participant reported “*I told my colleagues that it was important to calculate the drops per minute using a calculator”.*

In terms of the psychological determinants of practice, a number of participants felt that the CPD workshop had increased their capability to calculate drug doses: *“I learned some new calculations such as intravenous drug calculations”,* and others commented on increased motivation to ensure accuracy of drug calculations to prevent negative consequences of inaccurate calculations: “*I learned that it is important to calculate drugs properly because if not we can have hyper-dosage...which can cause resistance.*” Participants who had discussed the training with colleagues hoped that this would improve social opportunity within teams to calculate drug doses correctly. Generally, opportunity factors were seen as the key barrier to further implementing the training in practice. Some participants commented that staff shortages can cause time constraints which would limit their perceived opportunity to, for example, double check calculations or ask a colleague for help.

## Discussion

This article describes the development and initial evaluation of a drug calculation CPD training workshop in Beira Central Hospital and the application of behavioural science to refine the training to develop its impact and sustainability. Multi-disciplinary health professional partners from the UK and Mozambique including health psychologists iteratively developed the session. Applying evidence-based concepts and methods from behavioural science enabled the partners to include active learning activities and incorporate extra BCTs such as action planning and feedback. These have been shown to impact on professionals’ practice, [31] likely through developing participants’ perceived motivation, capability and perceived opportunity to put the training into practice. Additionally, working with local educators and equipping partners with physical resources hopefully helps the training to run sustainably in future and improve patient safety beyond the lifetime of the partnership. The UK partners are exploring similarly refining the drug calculation training delivered in local hospitals in Ipswich to nursing staff and other health professionals, so this work was an example of bidirectional learning between the partners in the UK and Mozambique.

Nurse self-rated confidence to calculate doses correctly was high both before and after training, even though teams struggled with some game questions reflecting previous studies into confidence-competence gaps in health professionals [29, 30] and potentially revealing social desirability bias among participants. However, there is little research into nurses’ drug calculation errors in practice in Mozambique or in the UK [32] so it may be that their confidence is well-founded. Participants’ intentions to use a calculator were also strong but significantly increased after the training (and having been provided with one). The qualitative data suggested the CPD workshop had been enjoyable for participants and pointed to areas where practice change was already happening following the workshop, as well as further opportunity barriers for senior staff to address.

There are limitations to this study. Firstly, we did not set out to directly compare the phase one with phase two of our training development, so did not collect the same data in both phases. As described, it was not possible to measure behaviours such as medication errors or health outcomes, nor long-term impact, given the limited data available and concerns from staff about the consequences of revealing difficulties and mistakes. Such challenges are a recognised barrier to medication error reporting and improvement across the globe. [8, 33, 34] This limits our ability to determine the impact of the CPD workshop itself and of the behavioural science input. However, part of the health psychologists’ role and impact was to begin to tackle this complex problem of evaluation. Self-reported measures of psychological determinants of behaviour were introduced as a more feasible and still behaviourally relevant proxy and their inclusion is a first step to evaluation. The game also provided opportunity to collect some objective competence data and promoted multi-disciplinary working between nursing and pharmacy colleagues in a relaxed, informal atmosphere. This is encouraging since medication safety is a multi-professional issue in which nurses represent ‘the final stage of defence’. [35] p.185 NHS England’s Medication Safety Officers Network also advocate the importance of learning from errors and developing a culture of openness [9] and the CPD workshop hopefully began this process.

Nevertheless, the evaluation data collection methods could be improved in future, as we relied on internal evaluation, opportunistic sampling, short translated and back-translated questions and responses, over a small timeframe. This may have resulted in some respondent and experimenter bias. It is always preferable to employ a local researcher from outside the partnership to collect data but time and budgetary constraints meant this was not possible. Further research of this kind would help demonstrate the effectiveness of medication safety training generally and more specifically using a behavioural science approach. Finally, it is clear that nurses’ drug calculation skills are only one factor affecting medication errors and medication safety as a whole. Some suggest that other medication safety skills are equally important when trying to mitigate the inherent risks associated with medicines [32].

## Conclusions

In conclusion, applying behavioural science helped a health partnership develop and begin to evaluate a CPD workshop. This included an inexpensive (around 90 USD) educational game facilitated by local healthcare educators which facilitated engagement in drug calculation training in a resource-poor setting. Additionally, as with many partnerships, there has been bidirectional learning with mutual benefit [36, 37].

These findings have wider implications for health partnerships. We would argue that the essential process of sharing insights, developing ideas, testing, reflecting and refining, and following behavioural science methods, was only possible through the support and trusting relationships built over several years of a health partnership scheme. The ongoing conversation facilitated through the partnership model enabled UK and Mozambique-based multi-disciplinary professionals, including health psychologists, to apply their expertise at each stage. Ultimately this led to a hopefully more sustainable health intervention with initial evidence of impact, in the form of a locally-owned, culturally-relevant and engaging training package to address identified global health needs.

## Abbreviations

BCH: Beira central hospital
BCT: Behaviour change technique
CPD: Continuing professional development
THET: The tropical health and education trust
